# Suppression of AKT-mTOR signal pathway enhances osteogenic/dentinogenic capacity of stem cells from apical papilla

**DOI:** 10.1186/s13287-018-1077-9

**Published:** 2018-11-29

**Authors:** Yosuke Tanaka, Soichiro Sonoda, Haruyoshi Yamaza, Sara Murata, Kento Nishida, Shion Hama, Yukari Kyumoto-Nakamura, Norihisa Uehara, Kazuaki Nonaka, Toshio Kukita, Takayoshi Yamaza

**Affiliations:** 1 Division of Oral Biological Sciences, Department of Molecular Cell Biology and Oral Anatomy, Kyushu University Graduate School of Dental Science, 3-1-1 Maidashi, Higashi-ku, Fukuoka, 812-8582 Japan; 2 Division of Oral Health, Department of Pediatric Dentistry, Growth & Development, Kyushu University Graduate School of Dental Science, 3-1-1 Maidashi, Higashi-ku, Fukuoka, 812-8582 Japan; 3 Kyushu University School of Dentistry, 3-1-1 Maidashi, Higashi-ku, Fukuoka, 812-8582 Japan

**Keywords:** Stem cells from apical papilla (SCAP), Phosphoinositide 3 kinase (PI3K), AKT, Mammalian target of rapamycin (mTOR), Scaffold-free spheroidal calcified construct

## Abstract

**Background:**

Stem cells from apical papilla (SCAP) are a subpopulation of mesenchymal stem cells (MSCs) isolated from the apical papilla of the developing tooth root apex of human teeth. Because of their osteogenic/dentinogenic capacity, SCAP are considered as a source for bone and dentin regeneration. However, little is understood about the molecular mechanism of osteogenic/dentinogenic differentiation of SCAP. Phosphoinositide 3 kinase (PI3K)-AKT-mammalian target of rapamycin (mTOR) signal pathway participates in regulating the differentiation of various cell types, such as MSCs. In this study, we examined the role of the PI3K-AKT-mTOR signal pathway in the osteogenic/dentinogenic differentiation of SCAP. Moreover, we challenge to fabricate scaffold-free SCAP-based spheroidal calcified constructs.

**Methods:**

SCAP were pretreated with or without small interfering RNA for AKT (AKT siRNA), PI3K inhibitor LY294402, and mTOR inhibitor rapamycin and were cultured under osteogenic/dentinogenic differentiation to examine in vitro and in vivo calcified tissue formation. Moreover, SCAP-based cell aggregates were pretreated with or without LY294402 and rapamycin. The cell aggregates were cultured under osteogenic/dentinogenic condition and were analyzed the calcification of the aggregates.

**Results:**

Pretreatment with AKT siRNA, LY294402, and rapamycin enhances the in vitro and in vivo calcified tissue-forming capacity of SCAP. SCAP were fabricated as scaffold-free spheroids and were induced into forming calcified 3D constructs. The calcified density of the spheroidal constructs was enhanced when the spheroids were pretreated with LY294402 and rapamycin.

**Conclusions:**

Our findings indicate that the suppression of PI3K-AKT-mTOR signal pathway plays a role in not only enhancing the in vivo and in vitro osteogenic/dentinogenic differentiation of SCAP, but also promoting the calcification of scaffold-free SCAP-based calcified constructs. These findings suggest that a suppressive regulation of PI3K-AKT-mTOR signal pathway is a novel approach for SCAP-based bone and dentin regeneration.

**Electronic supplementary material:**

The online version of this article (10.1186/s13287-018-1077-9) contains supplementary material, which is available to authorized users.

## Background

The stem cells that can be isolated from the apical papilla tissues of the developing tooth root apex of human permanent teeth, such as impacted third molars, are termed stem cells from apical papilla (SCAP) [1]. SCAP are identified as a population of postnatal mesenchymal stem cells (MSCs) with the capacity for self-renewal and multipotent differentiation into osteoblasts/odontoblasts, adipocytes, and neural cells. SCAP have the capacity to form de novo calcified tissues on hydroxyapatite/tricalcium phosphate (HA/TCP) carriers, when SCAP are subcutaneously transplanted into immunocompromised mice. Recent studies indicated that SCAP represent a promising resource for bone and dentin regeneration [1, 2]. However, little is understood about the molecular mechanisms involved in osteogenic/dentinogenic differentiation of SCAP.

Cranial neural crest cells (CNCCs) have a role in the development of the tooth root and its surrounding components including the root dentin, cementum, periodontal ligament, and alveolar bone proper. Functional integration of CNCCs during tooth root development is critically regulated by transforming growth factor beta (TGFβ)/TGF β receptor (TBR)-mediated signal pathways [3]. AKT, which is one of the non-canonical signals underlying TGFβ/TBR, is a phosphoinositide 3 kinase (PI3K)-dependent serine/threonine kinase and leads to activation of the mammalian target of rapamycin (mTOR) [4]. PI3K, AKT, and mTOR play specific roles in regulating cellular metabolism and differentiation of various cell types [5]. In stem cells from human exfoliated deciduous teeth (SHED), PI3K, AKT, and mTOR signals maintain stem cell properties (stemness) [6] and regulate the osteogenic/dentinogenic capacity [7, 8]. Given the post neural crest stem cell property of SCAP [9], the signals related to TGFβ/TBR are believed to participate in the maintenance of the stemness and regulation of the osteogenic/dentinogenic capacity of SCAP [10]. However, no study has reported the involvement of PI3K-AKT-mTOR signal pathway in the osteogenic/dentinogenic differentiation of SCAP.

Bone graft is the gold standard therapy for jawbone defects [11]. However, bone grafting may not be beneficial to the treatment of jawbone defects because of both the invasive surgical collection and donor shortage [12, 13]. Clinically available biomaterials including hydroxyapatite and β-tricalcium phosphate have improved bone defect healing. Recently, dental pulp stem cell (DPSC)-based grafts with biodegradable materials and/or apatite were developed for bone regeneration [14, 15]. However, bacterial infection and immune reactions during the biomaterial degradation are considered critical problems involved in the usage of biomaterials [16, 17]. To overcome these problems, various three-dimensional (3D) cell culture systems without scaffolds have been developed [18–20]. Spheroid culture is one of the 3D culture systems that does not utilize a scaffold [21–23] and is considered a potential tool for bone regeneration. However, little is known about the fabrication of SCAP-based scaffold-free spheroids. The calcification mechanism(s) of SCAP-based spherical constructs have also not been elucidated.

In this study, to understand the role of PI3K-AKT-mTOR signal pathway in the osteogenic/dentinogenic differentiation of SCAP, we examined the effect of the interfering RNA (AKT siRNA) for AKT and specific inhibitors for PI3K and mTOR, LY204402 and rapamycin, respectively. Moreover, we fabricated scaffold-free SCAP-based spherical calcified constructs and analyzed their calcification under the suppression of the PI3K-AKT-mTOR signal pathway.

## Methods

### Ethics statement and human subjects

Human permanent teeth samples were collected as discarded biological/clinical samples from healthy donors (20–23 years of age) in the Department of Pediatric Dentistry of Kyushu University Hospital. Procedures for handling the human samples were approved by the Kyushu University Institutional Review Board for Human Genome/Gene Research (Protocol Number: 393-01). We obtained written informed consent from the donors.

### Antibodies and probes

All the specific antibodies and probes used in this study were summarized in Additional file 1: Tables S1-S3.

### Isolation and culture of SCAP

Isolation and culture of SCAP were according to the previous study [1]. SCAP were isolated from the apical papilla tissue of the developing tooth root apex of extracted human impacted lower third molars of healthy donors as described in Additional file 2**:** Supplementary Methods. Attached colony-forming cells on plastic culture flasks were expanded. The growth medium consisted of 15% fetal bovine serum (FBS; Equitech-Bio, Kerrville, TX), 100 μM l-ascorbic acid 2-phosphate (Wako Pure Chemicals, Osaka, Japan), 2 mM l-glutamine (Nacalai Tesque, Kyoto, Japan), and premixed antibiotics containing 100 U/ml penicillin and 100 μg/ml streptomycin (Nacalai Tesque) in minimum essential medium Eagle alpha modification (αMEM; Thermo Fisher Scientific, Waltham, MA). The passage 3 (P3) cells were analyzed for determining the characterization as MSCs and SCAP according to previous reports [1, 24] and were used for further experiments.

### Population doubling (PD) assay

Cells were seeded on T-75 culture flasks with the growth medium. When the cells reached at a sub-confluent condition, the cells were passed and were sub-cultured. These culture steps were repeated until the cells lost their dividing ability. The PD score was calculated at every passage according to the equation: log_2_ (number of final harvested cells/number of initial seeded cells) [5]. The total score determined the PD scores. The PD scores were calculated in three independent assays per each dental pulp sample.

### Pretreatment with small interference RNA for AKT (AKT-siRNA) and inhibitors for PI3K and mTOR, LY294402 and rapamycin

For functional knock-down of AKT, SCAP were pretreated for 3 days with AKT-siRNA (20 nM; Santa Cruz Biotechnology, Santa Cruz, CA) in αMEM without antibiotics using Lipofectamine RNAiMax (Thermo Fisher Scientific) according to the manufacturer’s instructions. To block PI3K and mTOR signals, SCAP were pretreated for 3 days with specific inhibitors for PI3K and mTOR, LY294402 (50 μM, Wako Pure Chemicals) and rapamycin (100 nM, LKT Laboratories, St. Paul, MN), respectively. Both inhibitors were diluted in dimethyl sulfoxide (DMSO, Wako Pure Chemicals). For the controls, cells were treated with control scrambled siRNA (Santa Cruz Biotechnology) and DMSO (Wako Pure Chemicals) alone instead of AKT-siRNA and inhibitors for PI3K and mTOR. The pretreated SCAP were subsequently used for in vitro and in vivo osteogenic/dentinogenic analyses.

### In vitro osteogenic/dentinogenic assay

SCAP (P3, 100 × 10^3^) were grown to confluence on 60-mm dishes and were incubated in an osteogenic/dentinogenic medium. The osteogenic/dentinogenic medium was supplemented with 1.8 mM potassium dihydrogen phosphate (Merck, Kenilworth, NJ) and 10 nM dexamethasone (Merck) in the growth medium [1]. The osteogenic/dentinogenic medium was changed twice a week. As a control, SCAP were cultured in the growth medium. To analyze the expression of osteoblast-specific proteins and genes and intracellular signal molecules, the cultures were harvested 1 week after the induction and were used for western blot and reverse transcription quantitative polymerase chain reaction (RT-qPCR) analyses. To analyze the calcified nodule formation, the cultures were harvested 4 weeks after the induction and were used for Alizarin Red-S staining. To analyze the calcium content, the dye was extracted from Alizarin Red-S-stained samples and was measured according to a previous study [25].

### In vivo calcified tissue formation assay

In vitro calcified tissue formation was performed according to the previous studies [1, 25]. Briefly, SCAP (P3, 4 × 10^6^), which were cultured under the growth condition, were mixed with hydroxyapatite/tricalcium phosphate (HA/TCP) ceramic powders (40 mg, Zimmer Inc., Warsaw, IN). The mixture was implanted subcutaneously into the dorsal surface of Balb/cAJcl-*nu/nu* immunocompromised mice (male, 8-week-old; CLEA Japan, Tokyo, Japan). As the control, HA/TCP (40 mg, Zimmer Inc.) alone without SCAP was implanted. Eight weeks after the transplantation, the implants were harvested for histological assays. Some transplants were used for RT-qPCR analysis.

### In vitro fabrication of scaffold-free SCAP-based spherical calcified constructs

SCAP (P3) were seeded on low cell attachment PrimeSurface 96 U multiwell plates (Sumitomo Bakelite, Tokyo, Japan) at different densities (1 × 10^4^, 5 × 10^4^, 1 × 10^5^, 2 × 10^5^, 3 × 10^5^, 4 × 10^5^, 5 × 10^5^, and 1 × 10^6^ per well) and for different periods (1, 2, 3, 7, 14, 21, and 28 days) in the growth medium to form spheroids. The spheroids were subsequently cultured for 4 weeks in the osteogenic/dentinogenic medium. The medium was changed twice a week. As a control, some spheroids were cultured in the growth medium. At each period, the cultured spheroids were imaged with a microscopy. Only aggregated area was measured by ImageJ software (NIH), and non-aggregated area was ignored from the measurement. The surface of the spherical structures was observed under a stereo microscopy SteREO Discovery.V12 (Carl Zeiss Microscopy, Jena, Germany). The spherical structures were also used for microcomputed tomographic (micro-CT) analysis.

### Micro-CT analysis

Samples were imaged with a micro-CT scanning system Skyscan 1076 (Skyscan, Kontich, Belgium). Bone mineral density (BMD) and bone parameters were measured using CT-Analyzer (Skyscan) software as described previously [25]. BMD values were calibrated using hydroxyapatite phantoms with BMD values of 0.25 and 0.75 g/cm^3^ (Skyscan).

### X-ray fluorescence (XRF) analysis

SCAP-based spherical constructs were immersed in distilled water and were carefully cleaned with analytical grade ethanol. The calcium contents in the spheroids were analyzed on a JSX-1000S X-ray fluorescence spectrometer (JEOL, Tokyo, Japan) by the fundamental parameter method. As a control, a human deciduous tooth sample was used.

### Reverse transcription quantitative polymerase reaction (RT-qPCR) analysis

Total RNA was extracted and purified as described in Additional file 2**:** Supplementary Methods. The total RNA was used for preparing cDNA using a ReverTra Ace qPCR kit (TOYOBO, Osaka, Japan) according to the manufacturer’s instruction. The cDNA (10 μg) was applied for qPCR using EagleTaq Universal Master Mix (Roche, Basel, Switzerland) and target TaqMan probes (Thermo Fisher Scientific) on a Light Cycler 96 system (Roche). The PCR conditions were set up as the following: preincubation 1 (50 °C for 120 s), preincubation 2 (95 °C for 600 s), and two-step amplification (95 °C for 15 s and 60 °C for 60 s; 45 cycles). The target TaqMan probes were listed in Additional file 1**:** Table S2. The results of human 18S ribosomal RNA were used for normalization.

### Western blot analysis

Western blot analysis was performed as described in Additional file 2: Supplementary Methods. The specific antibodies used in the present western blot analysis were listed in Additional file 1**:** Table S2. The intensity of each band was measured by using an ImageJ (NIH) and was normalized with the intensity of the corresponding β-actin band as the internal control.

### Histological assay

Tissue samples were fixed with 4% paraformaldehyde in PBS overnight at 4 °C and were decalcified with 10% EDTA solution (pH 8.0). The samples were dehydrated and embedded in paraffin. Paraffin sections were treated with hematoxylin and eosin staining. For immunofluorescence, some paraffin sections were treated with primary antibody (Additional file 1**:** Table S3) and were incubated with Alexa Fluor 647-conjugated secondary antibody (Agilent, Santa Clara, CA). The sections were finally stained with DAPI (Nacalai Tesque). As an immunofluorescent control, sections were stained with isotype-matched antibody (mouse IgG_1_; Santa Cruz Biotechnology, Dallas, TX) instead of the primary antibodies. All sections were observed with a microscopy Axio Imager M2 (Carl Zeiss Microscopy, Jena, Germany). For morphological assay, seven fields were randomly selected from hematoxylin and eosin-stained sections. Newly formed mineralized tissue area in each field was measured by ImageJ software (National Institutes of Health [NIH], Bethesda, MA), and the ratios of mineralized tissue area over total tissue area were calculated.

### In vivo bone regeneration assay in a jawbone defect model

In vivo bone regeneration assay was performed in jawbone defect model mice [26]. The jawbone defect was prepared around the mesial buccal roots of left maxillary first molars of immunocompetent C57BL/6 mice (4 weeks old, SLC). SCAP or SCAP-based spheroids were pretreated with or without LY294402 (50 μM) and rapamycin (100 nM) for 3 days. The SCAP-based spheroids were subsequently cultured under osteogenic/dentinogenic condition for four weeks. The SCAP or SCAP-based spherical calcified materials (0.1 × 10^6^/spheroid) were implanted into the prepared bone cavities. The spherical calcified materials were pretreated with DNase (Promega, Fitchburg, WI) before the implantation. As a control for SCAP transplantation, HA/TCP alone was transplanted in the bone defects. As a control for SCAP-based calcified spheroids, no materials were implanted in the bone defects. The jaw bones implanted with or without SCAP-based spherical calcified materials were harvested 8 weeks after the implantation and were used for histological analysis. The bone defect area was histologically analyzed according to the previous criteria [26].

### Statistical analyses

The statistical results were expressed as the mean ± standard error of the mean (SEM) of at least triplicate determinations. Comparisons between two groups were analyzed by an independent two-tailed Student’s *t* test. Multiple group comparisons were performed by a one-way repeated measures analysis of variance followed by Tukey’s post hoc test. Values of *P* < 0.05 were considered to be significant. All the statistical analyses were performed using the PRISM 6 software program (GraphPad, Software, La Jolla, CA, USA).

## Results

### SCAP are multipotent MSCs

Isolated cells from the apical papilla tissues of human tooth roots had a capacity of forming adherent cell clusters on plastic dishes (Additional file 3: Figure S1a, S1b). The P3 cells express CD146, CD105, CD73, and CD90, but not CD34, CD45, and CD14 (Additional file 3: Figure S1c). The P3 cells also expressed CD24, which is a specific marker for undifferentiated SCAP [1] (Additional file 3: Figure S1c). The population doubling assay demonstrated a high cell proliferative ability of the P3 cells, but the ability was limited (total population doubling score, 76.5 ± 3.8). RT-qPCR analysis demonstrated that the P3 cells cultured under osteogenic/dentinogenic, chondrogenic, and adipogenic conditions significantly expressed the differentiation-specific genes (*runt related transcription factor 2* [*RUNX2*], *bone gamma-carboxyglutamate acid protein* [*BGLAP*], and *dentin sialophosphoprotein* [*DSPP*] for osteogenic/dentinogenic differentiation; *SRY-box 9* [*SOX9*] and *collagen type X alpha 1 chain* [*COL10A1*] for chondrogenic differentiation; *peroxisome proliferator-activated receptor gamma* [*PPARG*] and *lipoprotein lipase* [*LPL*] for adipogenic differentiation) in comparison with the corresponding non-induced control cells (Additional file 3: Figure S1d). This profiling indicated that our isolated cells from the apical papilla of human teeth were identified as SCAP based on the minimum criteria of MSCs [24] and the specific marker expression of SCAP [1].

### PI3K-AKT-mTOR signal pathway was upregulated in osteogenic/dentinogenic differentiation of SCAP

**Fig. 1:**
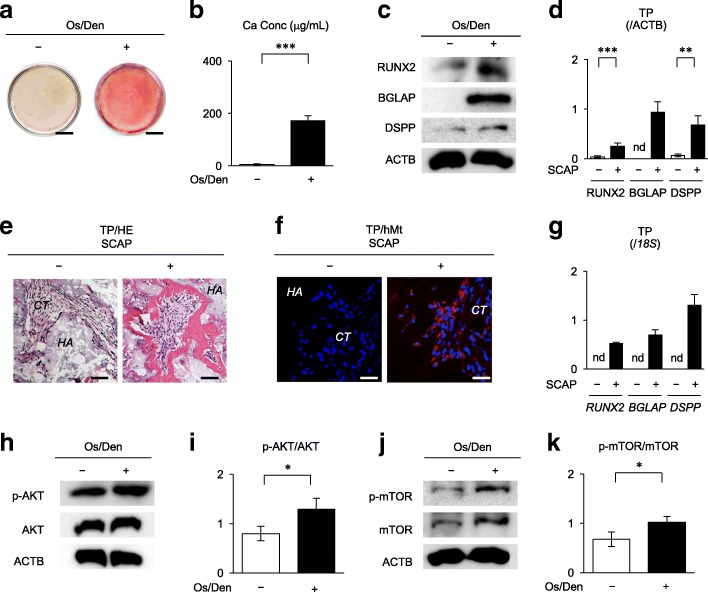
Osteogenic/dentinogenic differentiation of SCAP. **a**, **b** SCAP were cultured with or without osteogenic/dentinogenic medium (Os/Den) for 4 weeks. Alizarin Red-S staining shows the calcium deposition. Representative images of Alizarin Red-S staining. Bars = 25 mm (**a**). Calcium accumulation assay. ***P < 0.005. Graph bars show the means ± SEM (**b**). **c**, **d** SCAP were cultured with or without osteogenic/dentinogenic medium (Os/Den) for 1 week. Western blot analysis shows the expression of osteogenic/dentinogenic markers including runt-related nuclear factor 2 (RUNX2), bone gamma-carboxyglutamate protein (BGLAP), and dentin phosphoprotein (DSPP) in SCAP. Representative expression of RUNX2, BGLAP, and DSPP (**c**). Relative expression of RUNX2, BGLAP, and DSPP to β-actin (ACTB) (**d**). **e**–**g** In vivo calcified tissue formation assay was performed in immunocompromised mice using hydroxyl apatite/tricalcium phosphate (*HA*) as carriers with or without SCAP. Representative histological images of transplant tissues (TP) by hematoxylin and eosin staining (HE). Bars = 100 μm (**e**). Representative immunofluorescent images staining with anti-human mitochondria (hMt) antibody. Bars = 50 μm (**f**). RT-qPCR analysis shows the expression of *RUNX2*, *BGLAP*, and *DSPP* in the transplant tissues. Results shown as the ratios to the expression of the corresponding 18S ribosomal RNA (*18S*). nd, no detect (**g**). **h**–**k** SCAP were cultured with or without osteogenic/dentinogenic medium (Os/Den) for 1 weeks. Western blot analysis shows the phosphorylation of AKT and mammalian target of rapamycin (mTOR) in SCAP. Representative images of the expression of AKT, phosphorylated AKT (p-AKT), mTOR, and phosphorylated mTOR (p-mTOR) (**h**, **j**). Relative phosphorylation of AKT and mTOR (**i**, **k**). **b**, **d**, **g**, **i**, **k** *n* = 5 for all groups. *P < 0.05, ***P < 0.005. Graph bars show the means ± SEM. **e**, **f** *CT*, connective tissue; *HA*, hydroxyapatite/triphosphate

Alizarin Red-S staining showed that SCAP at 4 weeks after the osteogenic/dentinogenic induction showed a large amount of calcium deposition compared to non-induced control SCAP, which were cultured under the growth medium (Fig. 1a, b). Western blot analysis indicated that, at 1 week after the osteogenic/dentinogenic induction, the induced SCAP expressed markedly increased levels of RUNX2, BGLAP, and DSPP in comparison with the non-induced control SCAP (Fig. 1c, d). In vivo transplantation assay resulted in forming de novo calcified tissue formation in the SCAP-transplanted tissues (Fig. 1e). Immunofluorescent analysis demonstrated that human mitochondria-positive cells arranged along the de novo calcified matrix in the SCAP-transplanted tissues (Fig. 1f). No fluorescent intensity was found in any implant tissues stained with isotype-matched antibody instead of the primary antibodies (data not shown). RT-qPCR assay showed the expression of human *RUNX2*, *BGLAP*, and *DSPP* in the implant tissues with SCAP and HA/TCP (Fig. 1g). Meanwhile, no de novo calcified tissue and expression of human *RUNX2*, *BGLAP*, and *DSPP* was detected in the control implant tissues with HA/TCP alone (Fig. 1e–g).

Our preliminary RT-qPCR analysis demonstrated the expression of *TBR type I (TBR1)*, *TBR2*, and *TBR3* in SCAP (Additional file 3: Figure S2), suggesting that the intercellular signals underlying TGFβ/TBR may participate in SCAP function. Western blot analysis demonstrated that, at 1 week after the osteogenic/dentinogenic induction, the induced SCAP expressed a markedly enhanced phosphorylation of AKT and mTOR compared to the control non-induced SCAP (Fig. 1h–k). Meanwhile, phosphorylation of p38 and extracellular-signal-activated kinase 1 and 2 (ERK1/2) showed little difference between the osteogenic/odontogenic SCAP and the control non-induced SCAP (Additional file 3: Figure S3). These findings suggested that PI3K-AKT-mTOR signal pathway played a role in osteogenic/dentinogenic differentiation of SCAP.

### Suppression of PI3K-AKT-mTOR signal pathway accelerates in vitro osteogenic/dentinogenic differentiation of SCAP

**Fig. 2:**
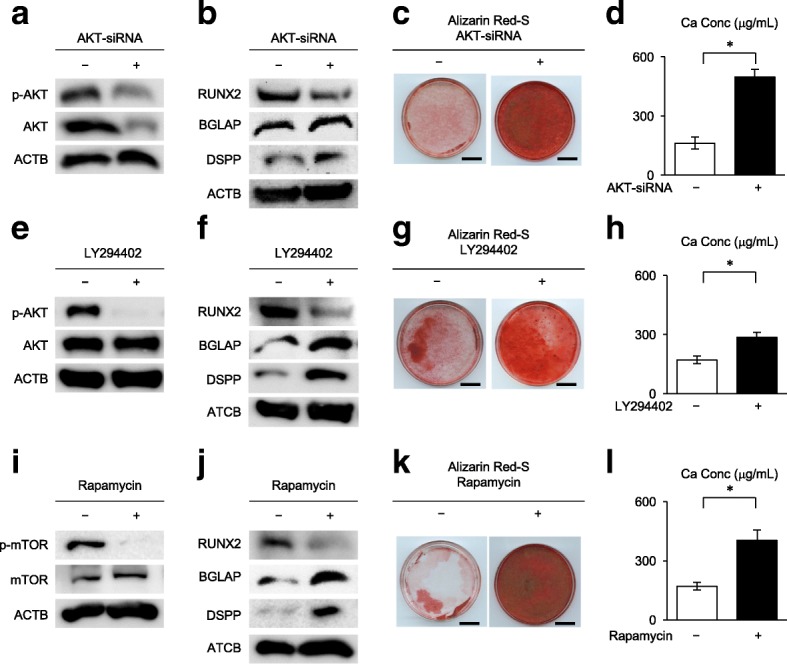
Effects of pretreatments with AKT-siRNA-, LY294402-, and rapamycin-pretreatment on in vitro osteogenic/dentinogenic differentiation of SCAP. SCAP were pretreated with or without AKT-siRNA (20 nM) (**a**–**d**), LY294402 (50 μM) (**e**–**h**), and rapamycin (100 nM) (**i**–**l**) for 3 days and were cultured under osteogenic/dentinogenic condition. **a**, **e**, **i** Representative western blot images of the expression of AKT and p-AKT (**a**, **e**) and mTOR and p-mTOR (**i**) in SCAP 1 week after the osteogenic/dentinogenic induction. **b**, **f**, **j** Representative western blot images of the expression of RUNX2, BGLAP, and DSPP in SCAP 1 week after the osteogenic/dentinogenic induction. **c**, **d**, **g**, **h**, **k**, **l** Alizarin Red-S staining shows the calcium deposition in SCAP 4 weeks after osteogenic/dentinogenic induction. Representative images of Alizarin Red-S staining. Bars = 25 mm (**c**, **g**, **k**). Calcium accumulation assay. *n* = 5 for all groups. *P < 0.05. Graph bars show the means ± SEM (**d**, **h**, **l**)

**Fig. 3:**
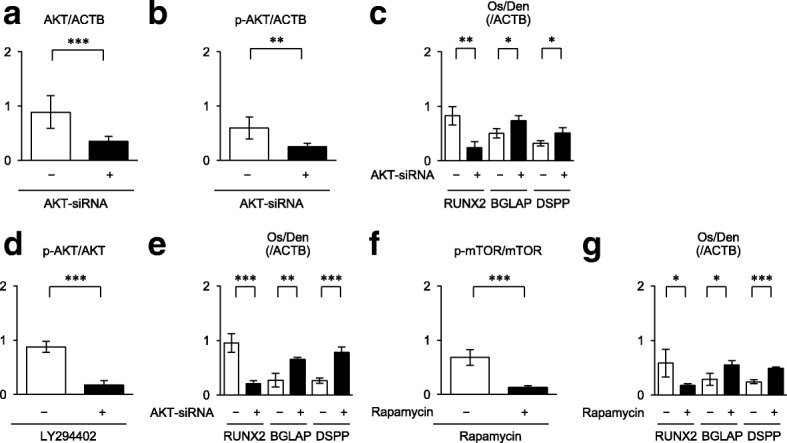
Phosphorylation of AKT and mTOR and expression of RUNX2, BGLAP, and DSPP in AKT-siRNA-, LY294402-, and rapamycin-pretreated SCAP under in vitro osteogenic/dentinogenic differentiation. SCAP were pretreated with or without AKT-siRNA (20 nM)- (**a–c**), LY294402 (50 μM)- (**d**, **e**), and rapamycin (100 nM) (**f**, **g**) and were cultured under osteogenic/dentinogenic condition (Os/Den) for 1 week. The expression of AKT, p-AKT, mTOR, p-mTOR, RUNX2, BGLAP, and DSPP was examined by western blot analysis and were shown in the Fig. 2a, b, e, f, i, j. **a**, **b**, **d**, **f** Relative expression of AKT/ACTB (**a**), p-AKB/ACTB (**b**), p-AKB/AKB (**d**), and p-mTOR/mTOR (**f**) in SCAP. **c**, **e**, **g** Relative expression of RUNX2, BGLAP, and DSPP in SCAP. **a**–**g** *n* = 5 for all groups. *P < 0.05, **P < 0.01, ***P < 0.005. Graph bars show the means ± SEM

To understand the role of PI3K-AKT-mTOR signal pathway in the osteogenic/dentinogenic differentiation of SCAP, SCAP were pretreated with AKT-siRNA (20 nM) 3 days before the osteogenic/dentinogenic induction (Additional file 3: Figure S3a). Western blot analysis confirmed the suppressive effect of AKT-siRNA pretreatment on AKT and phosphorylated AKT in SCAP (Figs. 2a, 3a, b). Of interest, western blot analysis demonstrated that the AKT-siRNA-pretreated SCAP exhibited a marked suppression of RUNX2 and significant enhancement of BGLAP and DSPP in comparison with control siRNA-pretreated SCAP 1 week after the induction (Figs. 2b and 3c). Alizarin Red-S staining 4 weeks after the induction showed that AKT-siRNA-pretreated SCAP exhibited markedly accelerated calcium accumulation in comparison with control siRNA-pretreated SCAP (Fig. 2c, d). SCAP were further pretreated for 3 days with the specific inhibitors for PI3K and mTOR, LY294402 (50 μM) and rapamycin (100 nM), respectively (Additional file 3: Figure S4a). LY294402- and rapamycin-pretreated SCAP were confirmed to exhibit reduced phosphorylation of AKT and mTOR, respectively, western blot analysis (Figs. 2e, i, and 3d, f). Western blot analysis demonstrated that both LY294402- and rapamycin-pretreated SCAP expressed the marked suppression of RUNX2 and significant enhancement of BGLAP and DSPP in comparison with the control SCAP (Figs. 2f, j, and 3e, g). Alizarin Red-S staining showed that both LY294402- and rapamycin-pretreated SCAP promoted enhanced calcium accumulation in comparison with the control SCAP (Fig. 2g, h, k, l).

### Suppression of the PI3K-AKT-mTOR pathway enhances SCAP-mediated in vivo calcified tissue formation

**Fig. 4:**
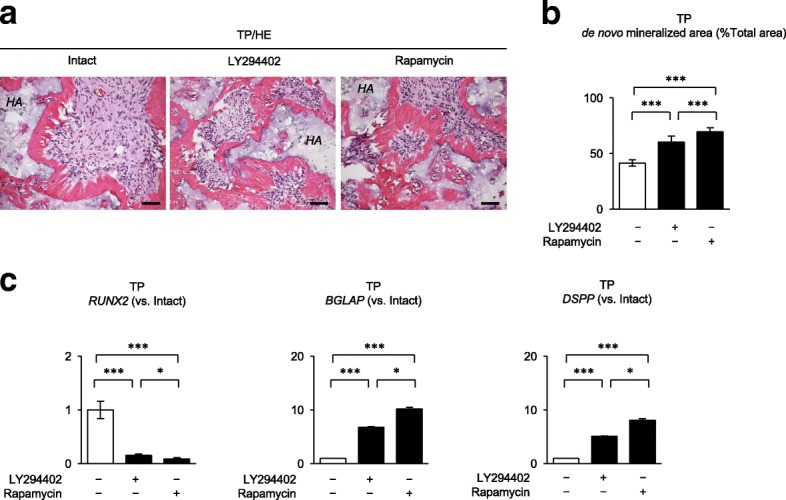
Effects of LY294402 and rapamycin on in vivo osteogenic/dentinogenic differentiation of SCAP. SCAP were pretreated with or without LY294402 (50 μM) and rapamycin (100 nM) and were subcutaneously transplanted into immunocompromised mice. **a** Representative images of the transplant tissues (TP) by hematoxylin and eosin staining (HE). *HA*, HA/TCP carriers. **b** Measurement of the area of de novo calcified tissues in the transplant tissues. **c** RT-qPCR analysis determines the expression of *RUNX2*, *BGLAP*, and *DSPP* in the transplant tissues. Results are shown as the ratios to the non-pretreated SCAP-transplant group. **a**, **c** Intact, non-pretreated SCAP-transplanted group. **b**, **c**: *n* = 5 for all groups. *P < 0.05 and ***P < 0.005. Graph bars show the means ± SEM

Next, LY294402 (50 μM)- and rapamycin (100 nM)-pretreated SCAP were subcutaneously transplanted (Additional file 3: Figure S4b). LY294402- and rapamycin-pretreated SCAP exhibited a significantly enhanced de novo formation of calcified tissues compared the control SCAP (Fig. 4a, b). RT-qPCR assay showed that the LY294402- and rapamycin-pretreated SCAP transplants showed the suppressed expression of human *RUNX2* and the enhanced expression of *DSPP* in comparison with the control SCAP transplants (Fig. 4c). Rapamycin-pretreated SCAP showed higher de novo calcified tissue formation, lower expression *RUNX2*, and higher expression of *BGLAP* and *DSPP* than LY294402-pretreated SCAP (Fig. 4).

### Fabrication of scaffold-free SCAP-based spherical calcified constructs

**Fig. 5:**
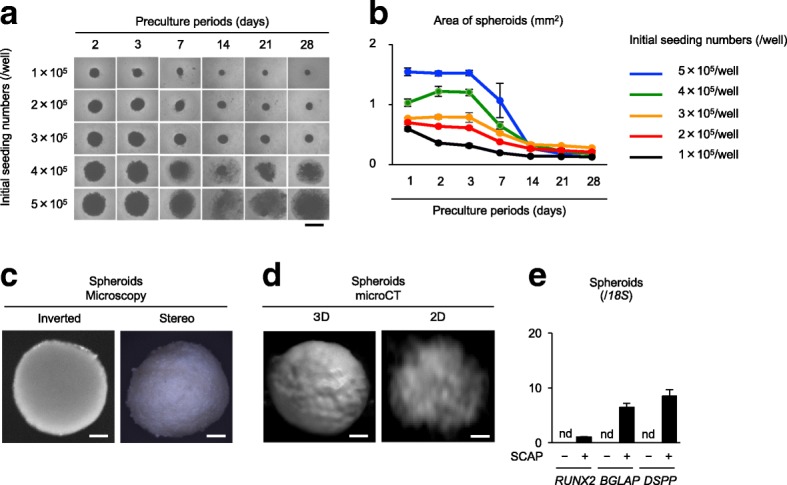
Fabrication of scaffold-free SCAP-based spherical calcified constructs. **a**, **b** Determination of the optimal requirement including initial seeding cell number and preculture period for SCAP spheroid formation. SCAP were seeded at the indicated initial number per well and were cultured for the indicated period. Representative microscopic images of each scaffold-free SCAP-based spheroids (**a**). Measurement of the area of the microscopic images of each scaffold-free SCAP-based spheroids (**b**). **c**–**e** Features of scaffold-free SCAP-based spherical calcified constructs. Scaffold-free SCAP-based spheroids were cultured under osteogenic/dentinogenic condition for 4 weeks. Representative inverted (left panel in **c**) and stereo (right panel in **c**) microscopic images and 3D (left panel in **d**) and tomographic (2D, right panel in **d**) micro-CT images of scaffold-free SCAP-based spheroidal calcified constructs. RT-qPCR analysis shows the expression of *RUNX2*, *BGLAP*, and *DSPP* in scaffold-free SCAP-based calcified constructs. Results show the ratios to the expression of the corresponding 18S ribosomal RNA (*18S*). nd, no detection (**e**). **a**, **c**, **d**: Bars = 1 mm (**a**), 100 μm (**c**, **d**). **b**, **e**: *n* = 5 for all groups. Graph bars show the means ± SEM

In our test, cell aggregates of SCAP (200 × 10^3^/well in 96-well multiwell plates), which were formed by 3-day preculture, were incubated for 4 weeks under osteogenic/dentinogenic condition. The aggregates showed differences in the size, shape, and calcification in each spheroid (data not shown). Some aggregates exhibited mutilated bodies. To determine the optimal conditions including initial seeding cell number per well in 96-well multiwell plates and pre-culture period for forming spheroids, SCAP were seeded at different numbers per well and were cultured for different periods. The seeded cells were aggregated once within 3 days after the seeding, some spheroid groups were mutilated (Fig. 5a, Additional file 3: Figure S5a). Based on the results from the preliminary tests (Fig. 5a, b**,** Additional file 3: Figure S5), we determined the optimal cell seeding number and pre-culture period to prepare spherical cell aggregates to 100 × 10^3^ per well and 7 days, respectively, in this study.

In the further study, spheroids were fabricated according to the determined optimal conditions (100 × 10^3^ per well, 7-day preculture) and were incubated under osteogenic/dentinogenic condition for 4 weeks. Morphological and micro-CT assays demonstrated that the spherical constructs expressed rough-surface spherical structures with heterogeneous electron-dense materials (Fig. 5c, d). No electron-dense material was detected in the control non-induced spheroids (data not shown). RT-qPCR determined the expression of *RUNX2*, *BGLAP*, and *DSPP* in the induced spheroids, but not in the control non-induced spheroids (Fig. 5e). Further XRF analysis detected calcium in the SCAP-based spheroids (*n* = 5, average contents 20.8 wt%), but not in control non-induced spheroids (*n* = 5, average contents 0 wt%). The average calcium content in a deciduous tooth as a control was 63.5 wt%.

### Pretreatment of SCAP-based spheroids with LY294402 and rapamycin enhances the calcification of SCAP-based spherical constructs

**Fig. 6:**
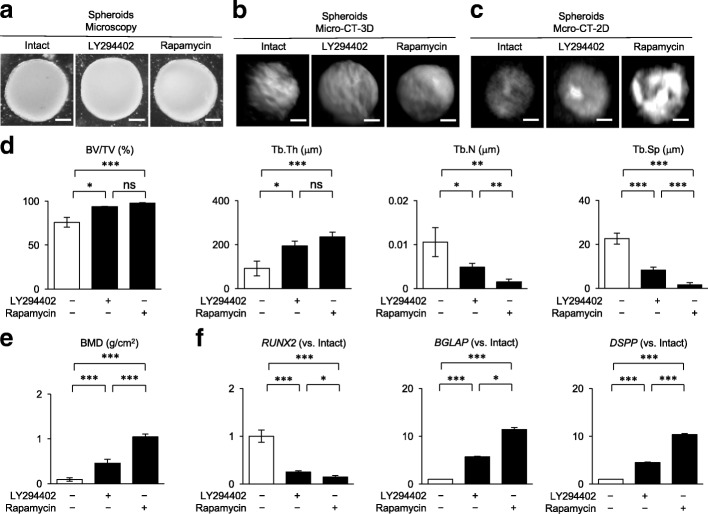
Effects of LY294402 and rapamycin on in vitro calcification of scaffold-free SCAP-based spheroids. SCAP-based spheroids were pretreated with or without LY294402 (50 μM) and rapamycin (100 nM) and were cultured under osteogenic/dentinogenic condition for 4 weeks. **a**–**c** Representative microscopic images (**a**), 3D (micro CT-3D, **b**) and tomographic (microCT-2D, **c**) micro-CT images of SCAP-based calcified spherical constructs. **d**, **e** Micro-CT analysis shows bone parameters (BV/TV, ratio of bone volume [BV] per total volume [TV]; Tb.Th, trabecular thickness; Tb.N, trabecular numbers; Tb.Sp, trabecular separation) (**d**) and bone mineral density (BMD) (**e**). **f** RT-qPCR analysis shows the expression of *RUNX2* and *DSPP* in SCAP-transplant tissues. Results show the ratios to the non-pretreated SCAP-transplant group. **d**–**f**: *n* = 5 for all groups. *P < 0.05, **P < 0.01 and ***P < 0.005. Graph bars show the means ± SEM. **a**–**c**, **f** Intact, non-pretreated SCAP-transplanted group

LY294402 (50 μM)- and rapamycin (100 nM)-pretreated SCAP-based spheroids were examined by microscopic and micro-CT analyses (Fig. 6a–e). The LY294402- and rapamycin-pretreated SCAP-based spheroids showed higher electron density than the non-pretreated control SCAP-based spheroids (Fig. 6c–e). RT-qPCR assay demonstrated that the LY294402- and rapamycin-pretreated spheroids promoted the decreased expression of *RUNX2* and enhanced expression of *BGLAP* and *DSPP* in comparison with the control spheroids (Fig. 6f). The rapamycin-pretreated spheroids promoted higher calcification and showed lower expression *RUNX2* and higher expression of *BGLAP* and *DSPP* than the LY294402-pretreated spheroids (Fig. 6c–f). Further XRF analysis showed calcium in all the SCAP-based spheroid groups (*n* = 5, averaged calcium content 24.4, 39.3, and 51.4 wt% in non-pretreated, LY294402-pretreated, and rapamycin-pretreated spheroids, respectively).

### LY294402- and rapamycin-pretreated SCAP and SCAP-based spherical calcified constructs enhance the bone regeneration in the jawbone defect

**Fig. 7:**
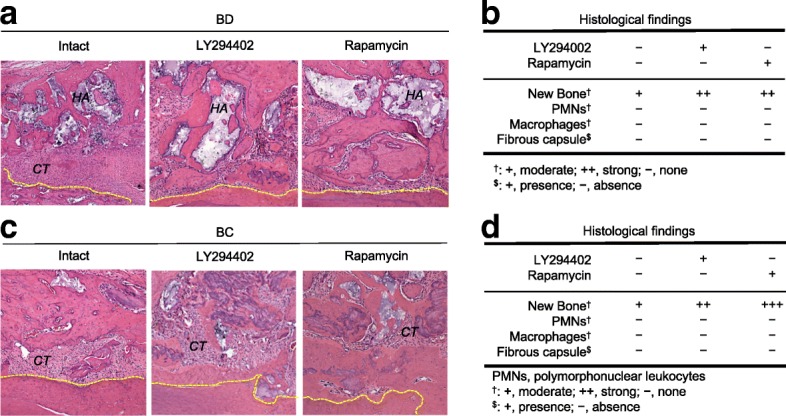
In vivo bone regeneration assay transplanted with SCAP and SCAP-based spherical calcified constructs. **a**, **b** SCAP were pretreated with or without LY294402 (50 μM) and rapamycin (100 nM) and were transplanted with hydroxyapatite/tricalcium carriers (*HA*) into the jawbone cavity of immunocompetent mice. Representative images of the jawbone cavity (BC) by hematoxylin and eosin staining. Intact, jawbone cavity transplanted with non-pretreated SCAP (**a**). Histological findings of jawbone cavity transplanted with SCAP-based spherical calcified constructs (**b**). **c**, **d** SCAP-based spheroids were pretreated with or without LY294402 (50 μM) and rapamycin (100 nM) and were cultured under osteogenic/dentinogenic condition for four weeks. The fabricated scaffold-free SCAP-based spherical calcified constructs were transplanted into the jawbone cavity of immunocompetent mice. Representative images of the jawbone cavity (BC) by hematoxylin and eosin staining. Intact, jawbone cavity transplanted with non-pretreated SCAP-based spheroids (**c**). Histological findings of jawbone cavity transplanted with SCAP-based spherical calcified constructs (**d**). **a**, **c** *CT*, connective tissue. Yellow-colored dot lines, original wall of bone cavity

SCAP and SCAP-based spheroids were pretreated with or without LY294402 and rapamycin. The SCAP-based spheroids were subsequently cultured under osteogenic/odontogenic condition. The SCAP were transplanted with HA/TCP carriers into jawbone defects of immunocompetent mice. The only calcified SCAP-based spherical constructs were transplanted into jawbone defects of immunocompetent mice. The bone defects transplanted with LY294402- and rapamycin-pretreated SCAP and SCAP-based spherical calcified constructs tended to form higher amount of de novo bone-like calcified tissue than that transplanted with non-pretreated SCAP and SCAP-based spherical calcified constructs (Fig. 7). Transplantation of the rapamycin-pretreated SCAP and SCAP-based constructs showed larger amount of de novo calcified tissue in the jawbone defect than that of LY294402-pretreated ones (Fig. 7). In the control groups transplanted with HA/TCP alone and no materials for SCAP and SCAP-based calcified spheroids, respectively, a little amount of de novo bone-like calcified tissue were formed in the bone defect (data not shown). Human mitochondria-positive cells were found along the de novo bone-like tissues in all bone cavities transplanted with SCAP pretreated with or without LY294402 and rapamycin (data not shown). All transplant tissues showed few inflammatory markers including invasion of polymorphonuclear cells and macrophages and formation of fibrous capsules (Fig. 7).

## Discussion

It was recently shown that activation of PI3K-AKT signaling promotes osteogenesis of human bone marrow-derived MSCs [27, 28]. In the present study, we demonstrated that PI3K-AKT signal pathway is activated in the osteogenic/dentinogenic differentiation of SCAP, but the pretreatment with LY294402 and AKT siRNA promotes the osteogenic/dentinogenic capacity of SCAP, suggesting the discrepancy in PI3K/AKT action between osteogenic/dentinogenic SCAP and osteogenic bone marrow-derived MSCs. Stage-specific regulation of early PI3K/AKT inhibition and late PI3K/AKT activation accelerates the osteogenic/dentinogenic differentiation of SHED [8]. Given the present finding using LY294003 and AKT-siRNA, the stage-specific activation of PI3K/AKT signaling may be a maturation switch required for osteogenic/dentinogenic differentiation of SCAP.

The present in vitro and in vivo studies showed that inhibition of PI3, AKT, and mTOR in SCAP increased the mineralized tissue-forming capacity. Of interest, the inhibition of PI3, AKT, and mTOR downregulated the expression of RUNX2 in SCAP after 1 week of the osteogenic/dentinogenic induction. Indeed, although the expression of *Runx2*, an essential transcriptional factor for the differentiation and maturation of osteoblasts [29], is constantly increasing during the differentiation of osteoblasts, the expression of *Runx2* is known to exhibit a stage-specific expression level of *Runx2* during the differentiation of odontoblast; the increased expression of *Runx2* in the early stage is suppressed in the terminal stage associated with the enhanced expression of DSPP [30, 31], suggesting that inhibition of PI3-AKT-mTOR signal pathway might accelerate the ability of osteogenic/dentinogenic differentiation of SCAP. Recent studies clarified that stage-specific regulation of the Runx2-targeting molecules, including microRNA 338-3p, microtubule-associated protein tau, small heterodimer partner, and Rho/Rho-associated protein kinase, transcriptionally suppresses *Runx2* expression, resulting in *DSPP* upregulation at the terminal stage of odontoblast differentiation [32–35]. Current studies report that AKT and its underlying signal FOXO1 negatively regulate Runx2 transcription in osteosarcoma and prostate cancer cells [36, 37]. The present negative regulation of PI3K-AKT-mTOR signal pathway in the osteogenic/dentinogenic differentiation of SCAP leads to speculation that PI3K-AKT-mTOR signal pathway transcriptionally may regulate RUNX2 expression in SCAP. Further study of the transcriptional regulation of *RUNX2* via the PI3K-AKT-mTOR signal pathway in SCAP will reveal the novel mechanism of SCAP-based osteogenesis/dentinogenesis.

In this study, we successfully fabricated scaffold-free SCAP-based spheroidal constructs and induced the SCAP-based spheroids into 3D calcified constructs associated with the gene expression of osteoblast-specific transcriptional factors and bone matrix proteins. Our SCAP-based constructs required no special machinery and complicated protocol for fabrication because of the usage of commercially available low attachment and U-bottomed multi-well-plates and general culture medium. Given the present transplant experiment in a jawbone defect, our fabricated scaffold-free SCAP-based calcified spherical constructs could be highly useful grafts for bone regeneration and may be used to overcome the critical problems involving bone grafts including the invasive surgical collection and donor shortage [12, 13], and the infection and inflammation caused by biomaterials [16, 17, 38]. Our fabricated scaffold-free SCAP-based calcified spherical constructs may have the potential for clinical application for dental implant therapy and endodontics. Further tissue-engineering-based preclinical studies may be necessary to clinically apply our fabricated scaffold-free SCAP-based calcified spherical constructs for regenerative medicine.

Culture systems in the 3D environment reconstruct the microenvironment and promote intracellular signaling activity in cell-cell interactions [39]. It was recently reported that 3D spheroid-cultured DPSCs exhibit a significant capacity for forming mineralized tissues compared with 2D monolayer-cultured DPSCs [16]. The 3D DPSC spheroids also consist of two zones: a cell-dense and well-calcified peripheral zone and cell-sparse and apoptotic core zone. mTOR signaling inhibits autophagy and induces apoptosis via inhibiting autophagy [40]. Of interest, in the present SCAP-based calcified constructs, rapamycin-pretreatment results in a marked mineral density *in vitro* and increased bone regeneration in vivo in comparison with LY294402-pretreatment. Since the inhibition of mTOR induces the osteogenic/dentinogenic differentiation of SHED at the early stages [8], it is indicated that we are capable of fabricating novel SCAP-based spheroidal constructs with a high mineral density, especially in the rapamycin-pretreated group. The present transplant study also suggests that SCAP-based spheroidal calcified constructs with higher mineral density might behave more osteoinductive in bone regeneration.

Animal models and 2D cell culture assays, which have implicated as alternative models for human organs/tissues, engage several problems such as high-cost experiments and drug responses in incorrect prediction of human responses by species-specific and structural-specific differences [41]. Given the direct multi-differentiation potency of SCAP [10, 42–44], it is suggested that the present scaffold-free engineered 3D-SCAP-spheroids can miniaturize human organs, resulting in implementing advantageous alternatives to the basic 2D screening platforms screening and current pre-clinical animal models. The scaffold-free engineered 3D-SCAP-spheroids may also be feasible functional 3D tissue buds to fabricate multiple human organs/tissues using a 3D bioprinter [45]. Further studies combined with the present scaffold-free engineered 3D spheroids will provide novel strategies in therapeutics of human diseases and high-throughput drug screening.

## Conclusion

The present findings suggest that PI3K-AKT-mTOR signal pathway is an important regulator of the osteogenic/dentinogenic differentiation of SCAP. The suppression of PI3K-AKT-mTOR signal pathway not only enhances the SCAP-based calcified tissue formation, but also promoted the calcification of scaffold-free SCAP-based calcified constructs, suggesting that a suppressive regulation of PI3K-AKT-mTOR signal pathway is a novel approach for SCAP-based bone and dentin regeneration. Further tissue-engineering-based preclinical studies will be necessary for SCAP-based bone and dentin regeneration.

## Additional files

Additional file 1: Supplementary Tables S1-S3. (PDF 154 kb)

Additional file 2: Supplementary Methods. (PDF 134 kb)

Additional file 3: Supplementary Figures S1-S5. (PDF 3490 kb)

## Abbreviations

BGLAP: Bone gamma-carboxyglutamic acid-containing protein
CNCCs: Cranial neural crest cells
DPSCs: Dental pulp stem cells
DSPP: Dentin sialophosphoprotein
FBS: Fetal bovine serum
MSCs: Mesenchymal stem cells
mTOR: Mammalian target of rapamycin
NF-κB: Nuclear factor kappa B
PI3K: Phosphatidyl inositol 3 kinase
RUNX2: Runt-related transcription factor 2
SCAP: Stem cells from apical papilla
siRNA: Small interfere RNA
TBR: Transforming growth factor beta receptor
TGFβ: Transforming growth factor beta
αMEM: Minimum essential medium alpha modification
