# Supplementary material for: Suppression of AKT-mTOR signal pathway enhances osteogenic/dentinogenic capacity of stem cells from apical papilla
Source: Stem Cell Res Ther. 2018 Nov 29;9:334. doi: 10.1186/s13287-018-1077-9 (PMC6264601; doi:10.1186/s13287-018-1077-9)
Supplement: Supplementary file 1 — Supplementary Tables S1-S3. (PDF 154 kb) [file 13287_2018_1077_MOESM1_ESM.pdf]

Supplementary Table 1. List of antibodies for flow cytometric analysis.

Names	Host/Isotype	Clones	Conjugate	Supplier
anti-CD14	mouse IgG_1, κ	61D3	R-PE	Thermo Fisher Scientific (Waltham, WA)
anti-CD24	mouse IgG_1, κ	SN3	R-PE	Thermo Fisher Scientific (Waltham, WA)
anti-CD34	mouse IgG_1, κ	4H11	R-PE	Thermo Fisher Scientific (Waltham, WA)
anti-CD45	mouse IgG_1, κ	2D1	R-PE	Thermo Fisher Scientific (Waltham, WA)
anti-CD73	mouse IgG_1, κ	AD2	R-PE	Thermo Fisher Scientific (Waltham, WA)
anti-CD90	mouse IgG_1, κ	5E10	R-PE	Thermo Fisher Scientific (Waltham, WA)
anti-CD105	mouse IgG_1, κ	SN6	R-PE	Thermo Fisher Scientific (Waltham, WA)
anti-CD146	mouse IgG1, κ	P1H12	R-PE	Thermo Fisher Scientific (Waltham, WA)

R-PE: R-phycoerythrin

Supplementary Table 2. List of TaqMan probes used for RT-qPCR

Name	Gene assay ID Number
BGLAP	Hs01587814_g1
COL10A1	Hs00166657_m1
DSPP	Hs00171962_m1
LPL	Hs00173425_m1
PPARG	Hs0115513_m1
RUNX2	Hs00231692_m1
SOX9	Hs01001343_g1
TBR1	Hs00232429_m1
TBR2	Hs00234253_m1
TBR3	Hs00234257_m1
Ribosomal RNA, 18S	Hs99999901_s1

BGLAP: *bone gamma-carboxyglutamate protein*

COL10A1: *collagen type X alpha 1 chain*

DSPP: *dentin sialophosphoprotein*

LPL: *lipoprotein lipase*

PPARG: *peroxisome proliferator-activated receptor gamma*

RUNX2: *runt related transcription factor 2*

TBR1: *transforming growth factor receptor type I*

TBR2: *transforming growth factor receptor type II*

TBR3: *transforming growth factor receptor type III*

Supplementary Table 3. List of specific antibodies for western blot and immunofluorescent analyses.

Name	Host/Isotype	Supplier
anti-ACTB	mouse IgG_1	Merck (Kenilworth, NJ)
anti-AKT	rabbit IgG	Cell Signaling Technology (Danvers, MA)
anti-AKT, phosphorylated (p-AKT)	rabbit IgG	Cell Signaling Technology (Danvers, MA)
anti-BGLAP	rabbit IgG	LifeSapn BioScience (Seattle, WA)
anti-DSPP	mouse IgG_{2b}	Santa Cruz Biotechnology (Dallas, TX)
anti-ERK1/2	rabbit IgG	Cell Signaling Technology (Danvers, MA)
anti-ERK1/2, phosphorylated (p-ERK1/2)	mouse IgG_1	Cell Signaling Technology (Danvers, MA)
anti-mitochondria, human	mouse IgG_1	Merck (Kenilworth, NJ)
anti-mTOR	rabbit IgG	Cell Signaling Technology (Danvers, MA)
anti-mTOR, phosphorylated (p-mTOR)	rabbit IgG	Cell Signaling Technology (Danvers, MA)
anti-p38	rabbit IgG	Cell Signaling Technology (Danvers, MA)
anti-p38, phosphorylated (p-p38)	rabbit IgG	Cell Signaling Technology (Danvers, MA)
anti-RUNX2	rabbit IgG	Abcam (Cambridge, England)

ACTB: actin, β

BGLAP: bone gamma-carboxyglutamate acid protein

DSPP: dentin sialophosphoprotein

ERK: extracellular signal-regulated kinases

mTOR: mammalian target of rapamycin

RUNX2: runt-related transcription factor 2
