# Supplementary material for: Suppression of AKT-mTOR signal pathway enhances osteogenic/dentinogenic capacity of stem cells from apical papilla
Source: Stem Cell Res Ther. 2018 Nov 29;9:334. doi: 10.1186/s13287-018-1077-9 (PMC6264601; doi:10.1186/s13287-018-1077-9)
Supplement: Supplementary file 2 — Supplementary Methods. (PDF 134 kb) [file 13287_2018_1077_MOESM2_ESM.pdf]

Supplementary Information

Suppression of AKT-mTOR signal pathway enhances osteogenic/dentinogenic capacity of stem cells from apical papilla

Yosuke Tanaka, Soichiro Sonoda, Haruyoshi Yamaza, Sara Murata, Kento Nishida, Shion Hama, Yukari Kyumoto-Nakamura, Norihisa Uehara, Kazuaki Nonaka, Toshio Kukita, Takayoshi Yamaza

Supplementary Methods

Isolation and culture of stem cells from apical papilla (SCAP)

Isolation and culture of SCAP were according to the previous study [Sonoyama et al., 2006] based on a colony-forming-unit fibroblast (CFU-F) method [Friedenstein, 1974]. Apical papilla tissues of extracted human permanent teeth (impacted lower third molars) were obtained from healthy donors. The tissue samples were digested with 0.3% collagenase type I (Worthington Biochemicals, Lakewood, NJ, USA) and 0.4% dispase II (Sanko Junyaku Co., Ltd., Tokyo, Japan) for 60 min at 37 °C. The obtained cells were passed through a 70-μm cell strainer and were seeded on culture flasks. Three hours after the cell seeding, the cultures were washed with sterilized Ca^{2+}-free and Mg^{2+}-free phosphate-buffered saline (PBS). The remaining adherent cells were incubated with a growth medium. The growth medium consisted of 15% fetal bovine serum (FBS; Equitech-Bio, Kerrville, TX), 100 μM L-ascorbic acid 2-phosphate (Wako Pure

Chemicals, Osaka, Japan), 2 mM L-glutamine (Nacalai Tesque, Kyoto, Japan), and premixed antibiotics (100 U/ml penicillin and 100 μg/ml streptomycin; Nacalai Tesque) in minimum essential medium Eagle alpha modification (αMEM; Thermo Fisher Scientific, Waltham, MA). Sixteen days after the seeding, attached colonies consisting of spindle-shaped cells were formed on the flasks and were passaged. The growth medium was changed twice a week. Passage 3 (P3) cells were analyzed for determining the profile as MSCs and SCAP according to the previous studies [Dominici et al., 2006; Sonoyama et al., 2006] and were used for further experiments.

CFU-F assay

Cells isolated from the apical papilla of permanent teeth were seeded on 100-mm dishes and were cultured for sixteen days. Attached colonies were treated with 2% paraformaldehyde and 2% toluidine blue and were washed with PBS.

Flow cytometric analysis

P3 cultured cells (0.1×10^6/100 μL) were suspended in ice-cold Hanks's balanced salt solution (HBSS) containing 2% heat-inactivated FBS (Equitech-Bio) and were incubated with R-phycoerythrin (R-PE)-conjugated target antigen-specific antibody (1 μg per antigen) at 4 °C for 45 min. As a control, R-PE-conjugated isotype-matched antibodies were used instead of the corresponding antigen-specific antibody. The stained samples were washed with HBSS containing 2% FBS (Equitech-Bio) and were analyzed on a flow cytometer FACSVerse (BD Bioscience, Franklin Lake, NJ). The number (percentage) of

positive cells was determined using a FACSuite software (BD Bioscience) by comparison with the corresponding control cells stained with the corresponding isotype-matched antibody, in which a false-positive rate of less than 1% was accepted [Yamaza et al, 2010]. The target antigen-specific antibodies used in the present flow cytometric analysis were listed in the Supplementary Table 1.

Multipotent differentiation assay

SCAP were induced in specific culture conditions for differentiating into osteoblasts/odontoblasts, chondrocytes, and adipocytes according to the previous studies [Sonoyama et al., 2006; Yamaza et al., 2011].

***In vitro* adipogenic induction assay**

Cultured SCAP (P3, 5×10^3/dish) were grown on 60-mm dishes in the growth medium until confluent and were cultured in an adipogenic medium supplemented with 500 μM isobutyl-methylxanthine (Merck, Kenilworth, NJ), 60 μM indomethacin (Merck), 0.5 μM hydrocortisone (Merck), and 10 μM insulin (Merck) in the growth medium. The adipogenic medium was changed twice a week. Four weeks after the induction, adipocyte-specific genes including peroxisome proliferator activated receptor gamma 2 (*PPARG2*) and lipoprotein lipase (*LPL*) were analyzed by reverse transcription quantitative polymerase chain reaction (RT-qPCR) assay. As a control, SCAP were cultured under the growth medium for four weeks.

***In vitro* chondrogenic induction assay**

Cultured SCAP (P3, 0.1×10^6/well) were aggregated in 96-well multiplates and were cultured in a chondrogenic medium. The chondrogenic medium contained 15% FBS (Equitech-Bio), 100 µM L-ascorbic acid 2-phosphate (Wako Pure Chemicals), 2 mM L-glutamine (Nacalai Tesque), 2 mM sodium pyruvate (Nacalai Tesque), 1% insulin-transferring-selenium mixture (Thermo Fisher Scientific), 100 nM dexamethasone (Merck), 10 ng/ml transforming growth factor β_1 (TGFβ_1; PeproTech, Rocky Hill, NJ, USA), and premixed antibiotics (100 U/ml penicillin and 100 µg/ml streptomycin; Nacalai Tesque) in Dulbecco's modified Eagle's medium (Thermo Fisher Scientific). The chondrogenic medium was changed twice a week. Six weeks after the induction, the chondrocyte-specific genes including SRY box 9 (*SOX9*) and collagen type X alpha 1 chain (*COL10A1*) were analyzed by RT-qPCR assay. As a control, SCAP were cultured under the growth medium for six weeks.

Extraction and purification of total RNA

Cell and tissues samples were treated with a total RNA extraction reagent TRIzol (Thermo Fisher Scientific) according to the manufacture's instruction. The extracted RNA samples were digested with DNase I (Promega) and were purified using an RNeasy Mini Kit (Qiagen, Venlo, Netherland). Contamination of genome DNA in the RNA extracts was determined as follows; the RNA extracts were directly reacted with a primer pair for human glyceraldehyde-3-phosphate dehydrogenase (Sense, 5'-

TGAAGGTCGGTGTCAACGGATTTGGC-3'; Antisense, 5'-CATGTAGGCCATGAGGTCCACCAC-3') and RT-PCR Quick Taq HS DyeMix on a T-100 thermal cycler (Bio-Rad Laboratories, Hercules, CA). The amplification by polymerase chain reaction (PCR) was as follows; initial activation step (95 ºC, 120 sec) and three-step cycling (95 ºC for 30 sec, 60 ºC for 30 sec, and 68 ºC for 60 sec; 30 cycles). The amplified PCR products (5 μl) were analyzed by 2% agarose gel electrophoresis. The gels were stained with ethidium bromide and were imaged on a Gel Doc EZ System (Bio-Rad Laboratories). Finally, no contamination of genome DNA in the extracted total RNA was confirmed.

Western blot analysis

Cultured SACP were lysed in M-PER mammalian protein extraction reagent (Thermo Fisher Scientific) supplemented with proteinase inhibitor cocktail (Nacalai Tesque) and phosphatase inhibitor PhoSTOP (Roche). They were separated by TGX FastCast acrylamide gels (Bio-Rad Laboratories) and were transferred on PVDF membranes (Bio-Rad Laboratories) using a Trans-Blot Turbo transfer system (Bio-Rad Laboratories). The membranes were blocked with 5% skim milk in Tris-buffered saline (150 mM NaCl and 20 mM Tris–HCl, pH 7.2) for 1 h at room temperature and were incubated with primary antibodies overnight at 4 °C and were treated with horseradish peroxidase (HRP)-conjugated secondary antibody (1:1000; Santa Cruz Biotechnology) for 1 h at room temperature. The membranes were visualized using SuperSignal West Pico (Thermo Fisher Scientific) on an Image Quant LAS 4010 imager (GE Healthcare Life Science,

Pittsburgh, PA). For the internal control assay, each membrane was treated with WB Stripping Solution Strong (Nacalai Tesque). The membranes were reprobed with anti-β-actin antibody (Merck), followed by incubating with HRP-conjugated secondary antibody (1:1000; Santa Cruz Biotechnology). The specific antibodies used in this study were summarized in the Supplementary Table 3.

References

[1] Sonoyama, W. et al. Mesenchymal stem cell-mediated functional tooth regeneration in swine. *PLoS ONE* **1**, e79 (2006).

[2] Friedenstein, A. J. et al. Precursors for fibroblasts in different populations of hematopoietic cells as detected by the in vitro colony assay method. *Exp Hematol* **2**, 83−92 (1974).

[3] Dominici, M. et al. Minimal criteria for defining multipotent mesenchymal stromal cells. The International Society for Cellular Therapy position statement. *Cytotherapy* **8**, 315–317 (2006).

[4] Yamaza, T. et al. Immunomodulatory properties of stem cells from human exfoliated deciduous teeth. *Stem Cell Res Ther* **1**, 5 (2010).

[5] Yamaza, T. et al. Mouse mandible contains distinctive mesenchymal stem cells. *J Dent Res* **90**, 317–324 (2011).
