# Supplementary material for: Suppression of AKT-mTOR signal pathway enhances osteogenic/dentinogenic capacity of stem cells from apical papilla
Source: Stem Cell Res Ther. 2018 Nov 29;9:334. doi: 10.1186/s13287-018-1077-9 (PMC6264601; doi:10.1186/s13287-018-1077-9)
Supplement: Supplementary file 3 — Supplementary Figures S1-S5. (PDF 3490 kb) [file 13287_2018_1077_MOESM3_ESM.pdf]

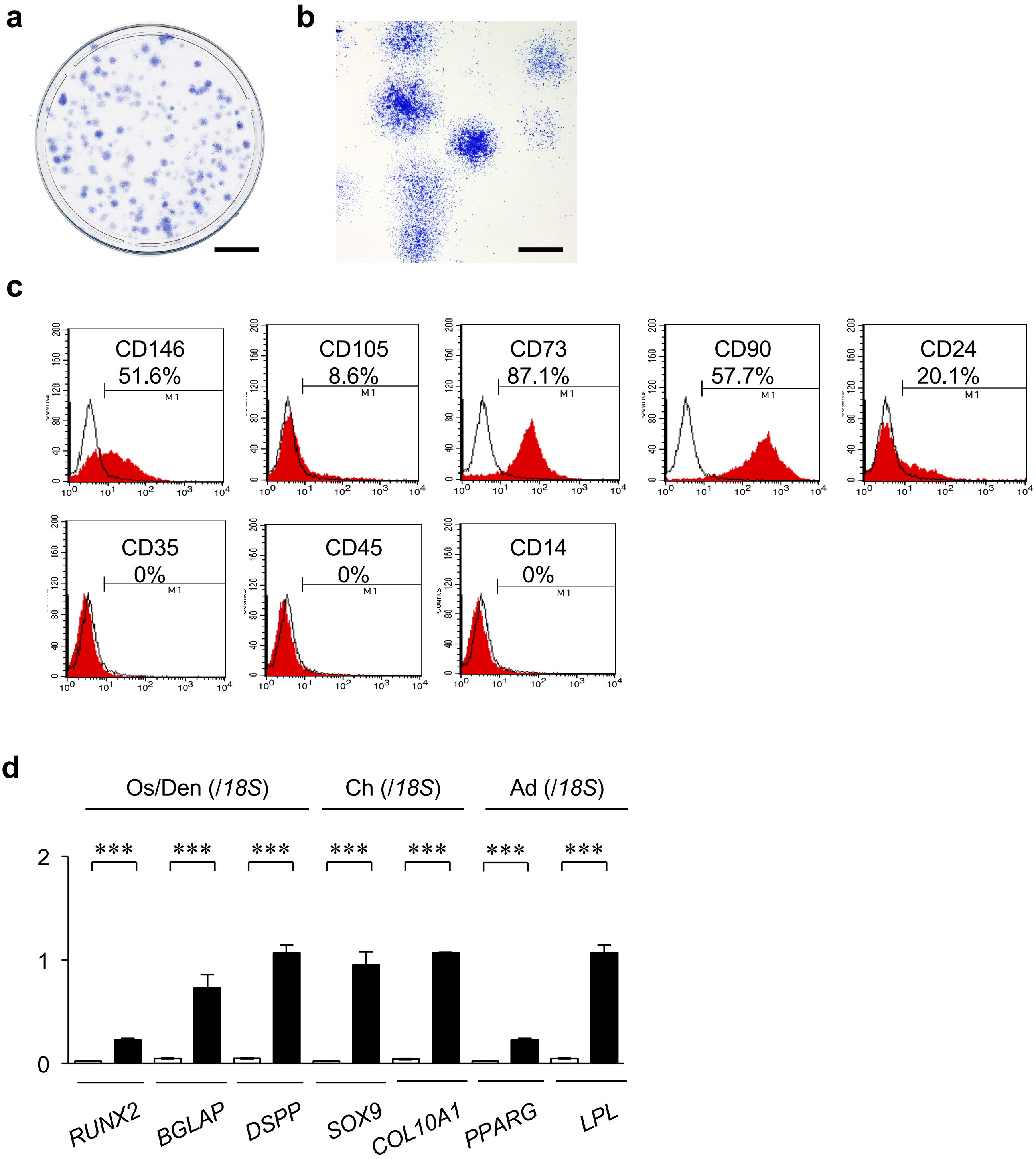

Supplementary Figure 1. Characterization of stem cells from apical papilla (SCAP). (a, b) Attached colony formation assay. Representative images of attached colonies (a) and different size and density of colonies (b). Bars = 20 mm (a), 5 mm (b). (c) Flow cytometric analysis shows the immunophenotype of SCAP. Representative histograms showing target-specific antibody-stained (red area) and isotype-matched antibody-stained cells (white area). Numbers show averages of positive rates. (d) SCAP were cultured under osteogenic/odontogenic (Os/Den), chodrogenic (Ch), and adipogenic (Ad) conditions. RT-qPCR analysis shows osteoblast/odontoblast, chodrocyte, and adipocyte marker genes in SCAP. Black columns, osteogenic/odontogenic, chodrogenic, or adipogenic SCAP; white columns, control SCAP. *BGLAP, bone gamma-carboxyglutamate acid protein; COL10A1*, *collagen type X alpha 1 chain*; *DSPP*, *dentin sialophosphoprotein*; *LPL*, *lipoprotein lipase*; *PPARG*, *peroxisome proliferator activated receptor gamma*; *RUNX2*, *runt related transcription factor 2*; *SOX9*, *SRY-box 9*. n = 5 for all groups. *** $P < 0.005$. Results show the ratios to the corresponding 18S rRNA (*18S*). Graph bars show the means ± SEM.

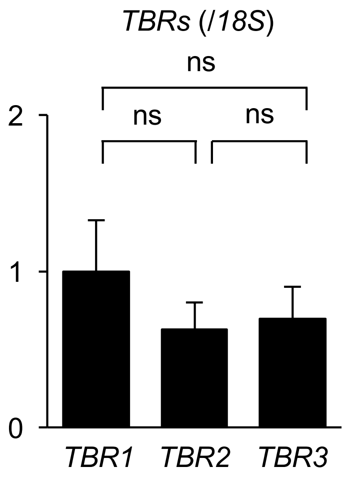

Supplementary Figure 2. Gene expression of transforming growth factor receptors (TBRs) in SCAP. RT-qPCR assay shows the expression of *TBR type I* (*TBR1*), *TBR2*, *and TBR3* in SCAP. Results show the ratios to the corresponding 18S rRNA (*18S*). n = 5 for all groups. ns: no significance. Graph bars show the means ± SEM.

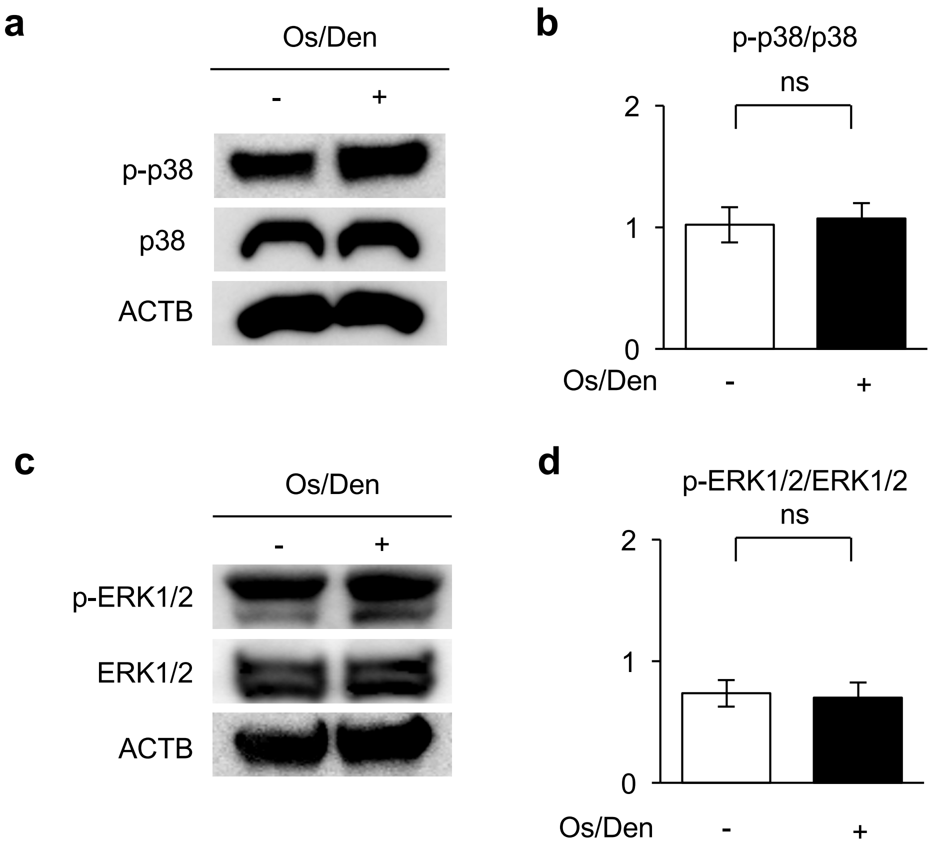

Supplementary Figure 3. Phosphorylation of p38 and ERK1/2 in the osteogenic/dentinogenic differentiation of SCAP. SCAP were cultured under osteogenic/dentinogenic condition (Os/Den) for 1 week. Western blot analysis shows the expression of p38, phosphorylated p38 (p-p38), extracellular-related kinase 1 and 2 (ERK1/2), and phosphorylated ERK1/2 (p-ERK1/2). (a, c) Representative images of the expression of p38 and phosphorylated p38 (p-p38) (a) and ERK1/2 and p-ERK1/2 (c). ACTB, beta-actin. (b, d) Relative expression of p-p38 to p-38 (p-p38/p38) (b) and p-ERK1/2 to ERK1/2 (p-ERK1/2/ERK1/2) (d). n = 5 for all groups. ns: no significance. Graph bars show the means ± SEM.

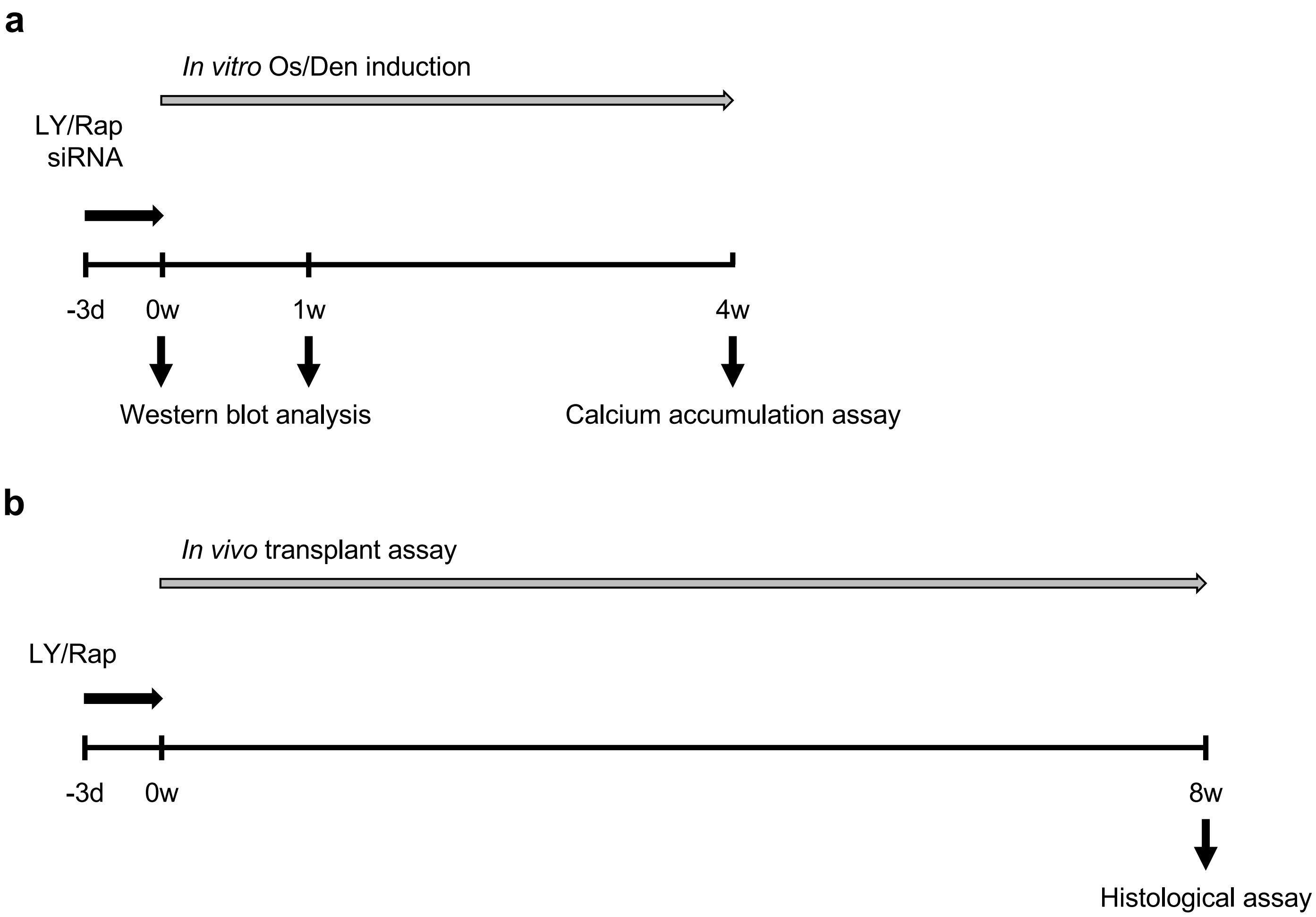

Supplementary Figure 4. Schemata of *in vitro* osteogenic/dentinogenic assay and *in vivo* transplant assay of SCAP. (a) A scheme of osteogenic/dentinogenic culture of SCAP. SCAP were pretreated with LY294402 (50 μM; LY), rapamycin (100 nM; Rap), and AKT siRNA (20 nM; siRNA) and were cultured under osteogenic/dentinogenic (Os/Den) condition. The cultures were harvested 0, 1, and 4 weeks after the osteogenic/dentinogeic induction for western blot analysis and calcium accumulation assay. (b) A scheme of *in vivo* transplantation assay. SCAP were pretreated with or without LY294402 (50 μM; LY) and rapamycin (100 nM; Rap) for 3 days. SCAP were subcutaneously transplanted with hydroxyapatite/tricalcium phosphate (HA/TCP) into immunocomromised mice. The transplants were harvested 8 weeks after the implantation and were used for histological assay.

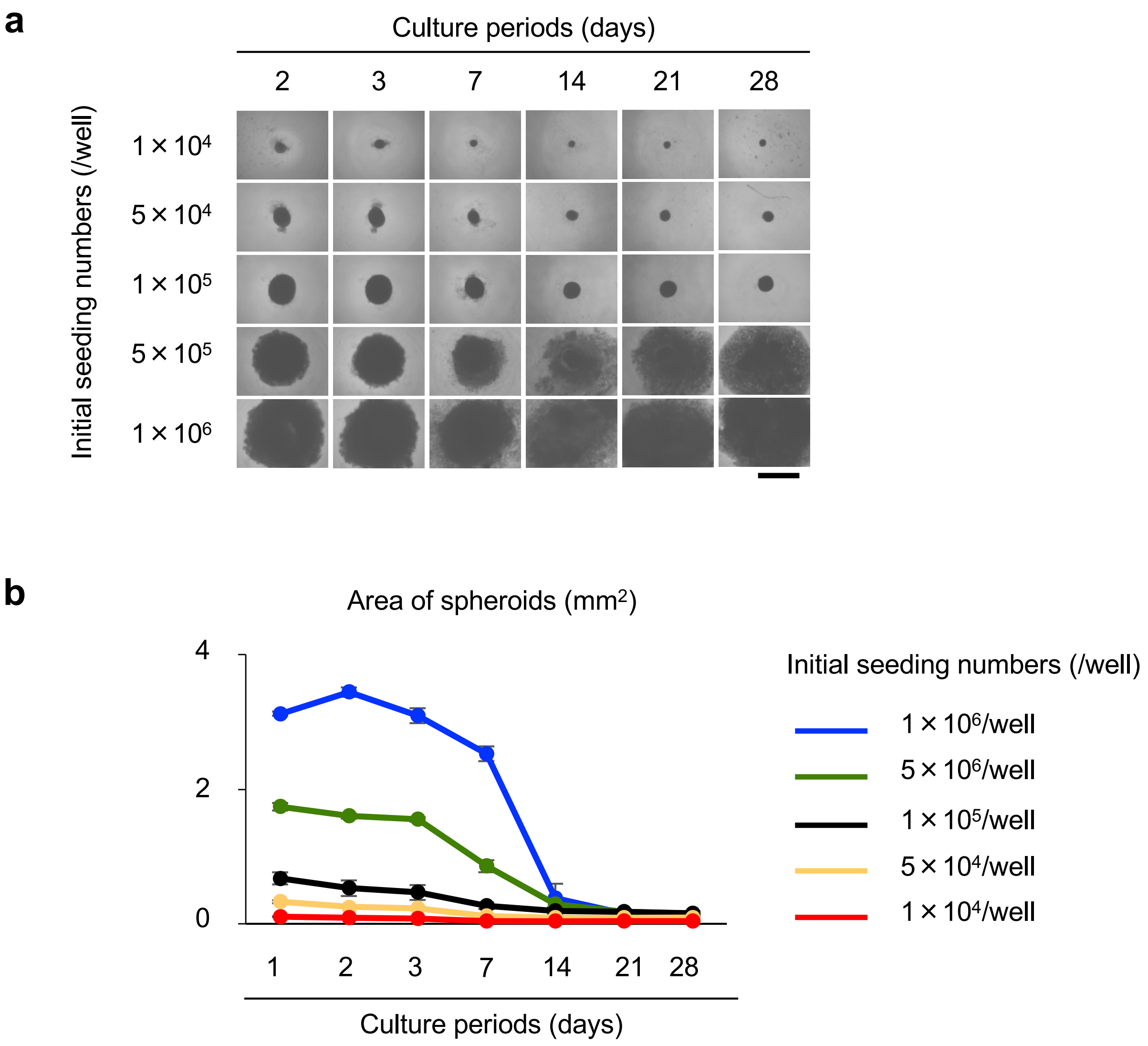

Supplementary Figure 5. Determination of the optimal requirement including initial seeding cell number and preculture period for SCAP spheroid formation. SCAP were seeded at the indicated initial number per well and were cultured for the indicated period. (a) Representative microscopic images of the SCAP-based spheroids. Some spheroids, especially spheroids initially seeded at 5×10^5 and 1×10^6 per well, were broken during the culture. (b) Measurement of the cell aggregated area of the microscopic images of SCAP-based spheroids. n = 5 for all groups. Graph bars show the means ± SEM (b).
